# Prevalence and factors associated with intrapartum and immediate postpartum obstetric complications in Ethiopia: a secondary analysis of a national longitudinal survey

**DOI:** 10.1080/16549716.2026.2754625

**Published:** 2026-11-03

**Authors:** Alehegn Bishaw Geremew, Claire T. Roberts, Shahid Ullah, Jacqueline H. Stephens

**Affiliations:** a Flinders Health and Medical Research Institute, College of Medicine and Public Health, Flinders University, Adelaide, South Australia; b Institute of Public Health, College of Medicine and Health Sciences, University of Gondar, Gondar, Ethiopia

**Keywords:** Maternal health, maternal morbidity, perinatal epidemiology, population-based survey, pregnancy outcomes

## Abstract

**Background:**

Most maternal deaths in Ethiopia occur during childbirth and the immediate postpartum period, yet population-level evidence on intrapartum and immediate postpartum complications and their determinants remains limited. In particular, the roles of intimate partner violence (IPV) during pregnancy and pregnancy complications have been underexplored.

**Objective:**

To determine the prevalence and factors associated with intrapartum and immediate postpartum obstetric complications in Ethiopia.

**Methods:**

We conducted a secondary analysis of the Performance Monitoring for Action Ethiopia (PMA-E) longitudinal survey (October 2019 and September 2020) using a two-stage stratified cluster sampling design. Multilevel modified Poisson regression analysis was used to estimate adjusted prevalence ratio (aPR) with 95% confidence intervals (CIs).

**Results:**

A total of 2558 women were included in the analysis. The prevalence of intrapartum and immediate postpartum complications was 37.6% and 31.6%, respectively. Rural residence (aPR = 1.72,95% CI: 1.34–2.20), intimate partner violence (IPV) during index pregnancy (aPR = 1.31,95% CI: 1.14–1.50), pregnancy complications (aPR = 1.78, 95% CI: 1.55–2.06), and nulliparity (aPR = 1.30, 95% CI: 1.07–1.58) were associated with intrapartum complications. Factors associated with immediate postpartum complications were complications during pregnancy (aPR = 1.65, 95% CI: 1.40–1.93) and prolonged labour (aPR = 1.34, 95%CI: 1.14–1.57). Childbirth at health facility was associated with a lower prevalence of immediate postpartum complications (aPR = 0.83, 95% CI: 0.70–0.98).

**Conclusion:**

Intrapartum and immediate postpartum obstetric complications are common in Ethiopia. IPV during pregnancy, pregnancy complications, and nulliparity were associated with intrapartum complications. Pregnancy complications and prolonged labour were associated with a higher prevalence of immediate postpartum complications, while health-facility childbirth was associated with a lower prevalence.

## Paper Context

- **Main findings**: This national population-based survey revealed a high burden of intrapartum and immediate postpartum obstetric complications in Ethiopia, and several factors significantly increased the likelihood of intrapartum complications and immediate postpartum obstetric complications.
- **Added knowledge**: This study provides national-level evidence on the burden of intrapartum and immediate postpartum complications in Ethiopia using population data; an approach rarely applied in low-resource settings and extends existing literature by identifying intrapartum- and immediate postpartum-specific factors associated.
- **Global health impact for policy and action**: The high prevalence of obstetric complications underscores the urgent need for strengthened intrapartum and postpartum care in low-resource settings, and findings underscore the need for policies to integrate intimate partner violence screening and mitigation strategies into routine antenatal and intrapartum care and strengthen antenatal care to identify women with pregnancy complication symptoms and provide further care for those women.

## Background

Despite considerable global progress in reducing maternal mortality over the last two decades [1], approximately 700 women die every day, which in 2023 equated to 260,000 maternal deaths worldwide, with 94% occurring in low- and middle-income countries (LMICs) [1,2]. These deaths are mostly due to preventable pregnancy-related causes [3,4]. Specifically, while Ethiopia has made remarkable progress (i.e. 72.6%) in reducing the maternal mortality ratio between 2000 and 2020 [5], it is still one of the nations with the highest number of maternal deaths (8,000 maternal deaths) annually [1]. According to the World Health Organisation 2025 report, Ethiopia’s maternal mortality ratio was 195 deaths per 100,000 live births in 2023 [1]. Furthermore, approximately 18% of women develop potentially life-threatening conditions during pregnancy, childbirth, or postpartum [6]. In resource-limited settings, failure to seek health care, delays, and failure to provide evidence-based interventions for obstetric complications contribute to high life-threatening complications and maternal deaths [6,7].

The intrapartum and immediate postpartum period are the highest risk window for severe maternal morbidity and mortality, in which most life-threatening complications occur [8]. In Ethiopia, two-thirds of maternal mortality occurs during the postpartum period [9], and among postpartum maternal mortality, half occur during the first 24 hours after childbirth [8]. Moreover, approximately 70–80% of deaths due to postpartum haemorrhage happen during the immediate postpartum period [8,10,11]. Therefore, the first 24 hours after childbirth are the most critical time for monitoring, early diagnosis, and timely intervention to prevent severe maternal morbidity and maternal deaths [12].

The three major obstetric complications (obstetric haemorrhage, hypertensive disorder of pregnancy, and sepsis) alone are responsible for more than half of maternal deaths globally [13]. Likewise, these causes contribute to more than 66% of maternal deaths in SSA [4]. Studies in Ethiopia reported direct obstetric causes account for 84–90% of deaths [3]. Furthermore, these maternal deaths are not mainly due to biomedical causes, but to determinants of maternal health and inequalities in healthcare use [14]. Despite the availability of evidence-based interventions for each major obstetric complication, mortality from these causes remains challenging [4]. Thus, focusing solely on the cause of mortality may be insufficient to improve maternal health; instead, preventing maternal deaths requires broad and proactive interventions that go beyond addressing the causes of death, which often occur in the late stages of maternal morbidity [15]. Efforts should focus on the entire continuum of obstetric morbidity and improving the quality of care, from maintaining well-being to early management of complications [14]. Evidence on complications across the maternity continuum is more beneficial than mortality alone in improving the health of both mother and newborn [16].

Different associated factors of obstetric complications have been addressed in the literature, such as age, residency, wealth status, parity, ANC, birth interval, previous caesarean section, and chronic medical disease [17–21]. However, these associated factors may differ for obstetric complications across the maternity continuum (pregnancy, childbirth, and the postpartum period). Furthermore, evidence remains limited on associated factors unique to pregnancy, childbirth, and postpartum obstetric complications.

Information on obstetric complications and specific factors associated with each pregnancy, childbirth, and postpartum complication is valuable for intervention development, especially in Ethiopia, where potential life-threatening maternal complications are prevalent [6]. However, most existing evidence combines measurements of obstetric complications occurring during pregnancy, the intrapartum period, and the postpartum period together as obstetric complications [17,21]. This approach may obscure differences in factors associated, as some determinants of obstetric complications vary across the maternity continuum. Furthermore, clinically and epidemiologically, some of the intrapartum complications are themselves factors associated with postpartum complications. For example, prolonged labour is strongly associated with severe postpartum complications [18,19]. These reasons warrant explicit investigation of the link between intrapartum and immediate postpartum complications.

Most available studies in Ethiopia identified obstetric complications among women who visited health facilities [17,22,23] or in a limited geographical area [24]. Acknowledging that facility-based data may be less prone to recall bias and measurement, this data are less representative, particularly in Ethiopia, where more than half of women give birth at home, more than two-thirds do not use postnatal care (PNC), and there is inadequate use of antenatal care (ANC) [25]. As such, evidence generated in health facilities may not be generalisable to the general pregnant, labouring, and postnatal women in Ethiopia. Additionally, in previous studies, participants were interviewed within the year after delivery [26]; recall bias becomes a significant issue as the time gap between the occurrence of obstetric complications and the interview increases. Women may forget key details of their complications, leading to underreporting. Hence, interviews should be conducted soon after birth for self-reported complications, as this allows women to recall and report their experiences with reasonable accuracy [27]. Existing population-based studies that interviewed women within 6 weeks postpartum have not addressed factors associated with complications [28], immediate postpartum obstetric complications, and important factors associated with intrapartum complications; for example, IPV during pregnancy and complications during pregnancy were not addressed [24]. Therefore, this study aimed to investigate the prevalence and factors associated with intrapartum and immediate postpartum obstetric complications among postpartum women in Ethiopia.

## Methods

### Study design and data source

We conducted a secondary analysis of national longitudinal cohort data collected at baseline and 6-week postpartum follow-up between October 2019 and September 2020. This study used data from the Performance Monitoring for Action (PMA) Ethiopia longitudinal survey. The PMA Ethiopia longitudinal survey was conducted through a partnership between Addis Ababa University, the Ethiopian Federal Ministry of Health, and the Johns Hopkins Bloomberg School of Public Health. The PMA Ethiopia longitudinal survey was conducted in five large regions of Ethiopia, including Tigray, Afar, Amhara, Oromia, South Nations, Nationalities, and Peoples (SNNP), and Addis Ababa city administration. In the PMA Ethiopia longitudinal survey, a two-stage stratified cluster sampling procedure was employed to select participants. Pregnant and postpartum women were recruited for a longitudinal survey, and follow-up interviews were conducted at 6 weeks, 6 months, and 1 year after delivery. The detailed survey design, sampling methods, and data collection of the PMA longitudinal survey are available and published elsewhere [29]. We obtained the dataset from the PMA website after receiving authorisation via an online request, stating the purpose of our research [30,31].

During the baseline survey at enrolment, sociodemographic characteristics, including residency, region, household wealth status, employment, education status, gestational age, history of family planning use, pregnancy intention, preconception care, community norms regarding ANC, and facility birth were collected. At the 6-week postpartum follow-up interview, data were collected on the utilisation of ANC, receipt of ANC packages, intimate partner violence (IPV) during pregnancy, obstetric complications (during pregnancy, labour and childbirth, immediate postpartum), mode of childbirth, birth assistance, and birth outcome.

This study used data from the baseline and 6-week postpartum follow-up surveys. All women enrolled during pregnancy or postpartum who had given birth were included. Of 2,855 enrolled participants in the baseline survey, 2665 completed the 6-week postpartum follow-up interview, while 190 women were not interviewed for various reasons. In addition, pregnant women who enrolled while pregnant and ended up with a termination of pregnancy (100) or where the outcome was not recoded (7) were excluded. As a result, a total of 2,558 postpartum women were included in the analysis. For stratified analysis, 13 participants who responded ‘I don’t know’ to intrapartum complications and 27 participants who responded ‘I don’t know’ to immediate postpartum complications were excluded (Figure 1).

**Figure 1.**
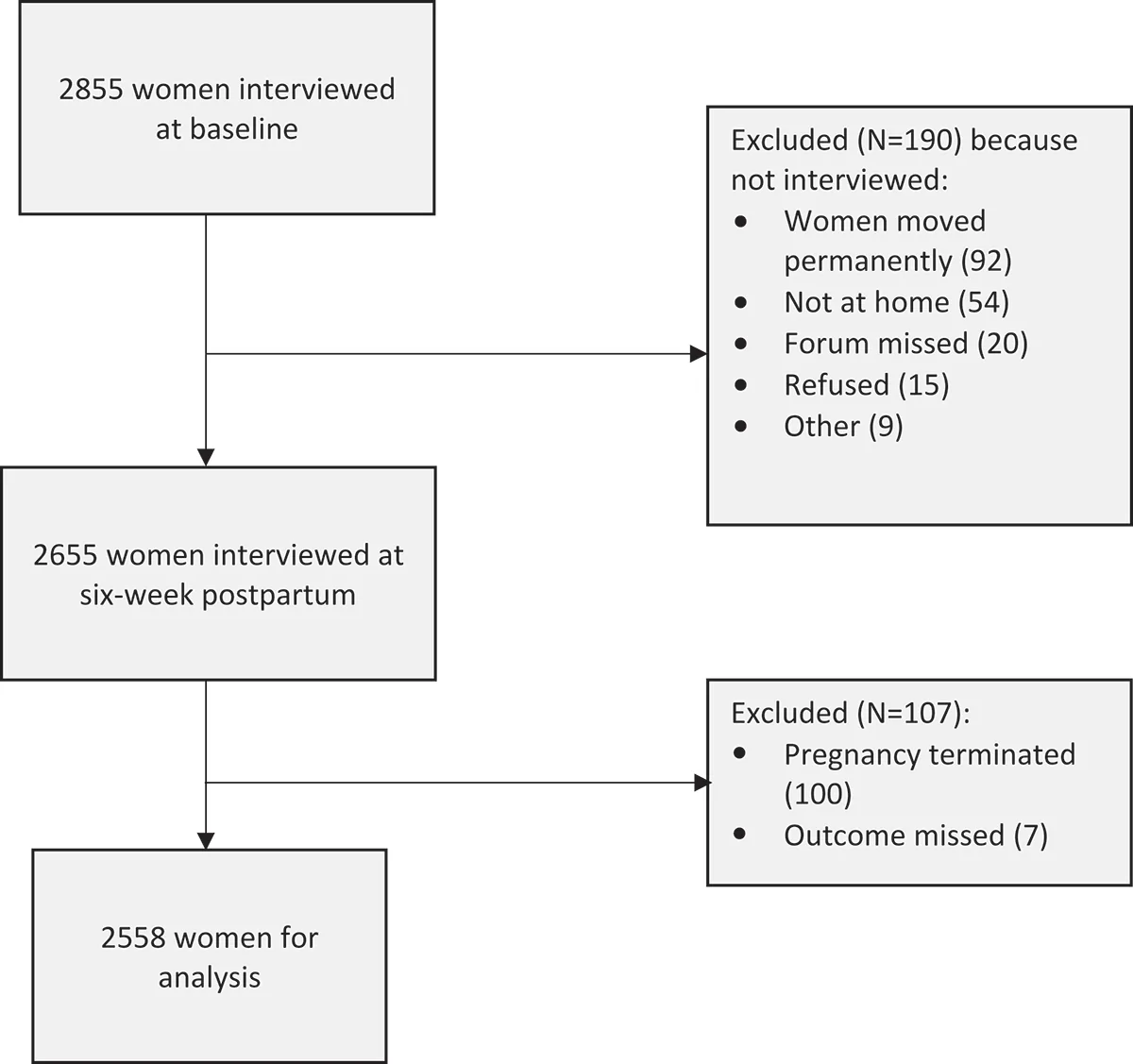
Selection of participants for prevalence and factors associated with intrapartum and immediate postpartum obstetric complications using the PMA Ethiopia baseline and 6-week postpartum survey data.

### Study variables

#### Outcome variables

Intrapartum obstetric complications were considered if women reported one of the following: excessive vaginal bleeding during labour, premature and prolonged rupture of membranes (PPROM), malpresentation/positions (the fetal part engaging in the maternal pelvis is other than the head), prolonged labour (>12 hours) and convulsions/fits [27,28,32]. Premature and prolonged rupture of membranes was defined as membrane rupture and no labour pain for more than 24 hours.

An immediate postpartum obstetric complication was defined as a complication that occurred within 24 hours after childbirth. Women were considered to have an immediate postpartum obstetric complication if they reported one of the following four complications: retained placenta for more than 30 minutes, high fever with foul/smelly discharge, excessive bleeding (postpartum haemorrhage), and convulsions/fits [27,28]. A recent Delphi consensus study by Dey T. et al, concluded that the symptoms of these obstetric complications can be self-assessed by women and birth companions [33].

#### Independent variables

Independent variables were selected based on the literature and the availability in the datasets. We considered socio-demographic, reproductive, and maternal health service variables. These variables were grouped into individual‑level and community‑level factors. Individual‑level variables included age, religion, marital status, educational status, household wealth status, preconception care, IPV during pregnancy, pregnancy intentions, complications during pregnancy, parity, ANC, and place of childbirth. Community-level variables included region, place of residence, and community-level use of maternity waiting homes.

Preconception care was defined as women reporting at least one of the following actions before pregnancy: seeking medical care/health advice, taking folic acid/multivitamins, or engaging in dietary modification by eating healthier foods.

Pregnancy intention was measured by asking women: ‘At the time you became pregnant, did you want to become pregnant then, did you want to wait until later, or did you not want to have any/any more children at all?’ Responses included ‘then,’ ‘later,’ and ‘not at all.’ Following a previous study, a binary variable was created in which ‘then’ was coded as intended (0) and ‘later’ or ‘not at all’ were coded as unintended [34].

IPV during pregnancy was defined as experiencing at least one physical or sexual act of violence from an intimate partner during the index pregnancy [35,36].

Complications during pregnancy were defined as a woman reporting at least one of the following symptoms during the index pregnancy: vaginal bleeding, high fever, convulsions/fits, severe headache with blurred vision, lower abdominal pain/abnormal vaginal discharge, high blood pressure, and oedema of the face/body.

Household wealth status was assessed using household assets, livestock ownership, household construction materials, drinking water sources, and sanitation facilities, and a wealth index generated using principal component analysis. Finally, the wealth score was categorised into five quartiles.

### Data management and statistical analysis

Data management and statistical analyses were performed using Stata version 18 (StataCorp LP, College Station, TX, USA) [37]. Missing outcome variables due to failure to record observations (7 observations) were deleted because this was assumed to be missing completely at random. Missing independent variable data (0.5%) where it was assumed missingness was at random were managed with simple imputations (mean or mode), as the missingness was small (<5%) multiple imputation was not considered necessary and did provide meaningful benefit [38].

Sample weights were applied to correct for the complex sampling design and non-response rate, ensuring that the analysis reflects the broader population. In the PMA Ethiopia longitudinal survey dataset, a sample weight variable was constructed to account for enumeration area selection probabilities, household selection, and women’s response rates. We used the Stata command *aweight* for descriptive analysis and *pweight* for multilevel modelling analysis. In the frequency analysis, ‘don’t know’ responses for complication status were included to avoid inappropriate coding of participant responses as either yes or no. Variable inflation factors (VIF) for immediate postpartum complications were < 5, and the mean VIF was 1.75.

Intrapartum and immediate postpartum obstetric complications within and between clusters were assessed using the intra-class correlation coefficient (ICC) and the median prevalence ratio (MPR). ICC is the proportion of total variation explained by the cluster. When selected at random, the MPR represents the average prevalence ratio between women in high-complication clusters and those in low-complication clusters. MPR was calculated as MPR = exp(0.95*σ2) which provided a meaningful measure of variance [39,40]. Initially, the intra-class correlation coefficient (ICC) was calculated using a multi-level logistic regression intercept-only model. When the outcome prevalence/incidence is less than 10%, logistic regression can overestimate the effect estimate (odds ratio), and modified Poisson regression can better estimate the effect for a binary outcome [41,42]. Hence, a modified Poisson regression model with robust standard errors was employed. Multilevel modelling was employed to account for clustering and the hierarchical nature of the data (Stata command syntax provided in supplementary file). Four models were applied: Model 1 (intercept-only model), Model 2 (individual-level variables), Model 3 (community-level variables), and Model 4 (both individual- and community-level variables). Model comparison was based on the Akaike information criterion (AIC), with the lowest value considered the best. Effect estimates were reported using aPRs with 95% CI; statistical significance was declared at *p* < 0.05.

### Ethical considerations

Ethical approval to use de-identified datasets for this study was received from the Flinders University Human Research Ethics Committee (Ref: HREC-6274) in July 2024. Data access was requested from the PMA data lab at the Johns Hopkins School of Public Health (JHSPH) via https://www.pmadata.org/data/available-datasets/request-accessdatasets, and approval was granted. The PMA Ethiopia longitudinal survey was conducted in accordance with the Declaration of Helsinki for research involving humans.

## Results

### Characteristics of participants

Table 1 presents the characteristics of the participants; 2,558 postpartum women were included in the analysis. The mean age of participants was 27.3 years (SD ±6.11 years). Approximately one-third of the participants (30.2%) were in the 25–29 age group. More than three-fourths (77.3%) of the participants were rural residents, and nearly all (97.7%) were married. More than 2 out of every 5 women (41%) had never attended any school. Nearly one-fifth (18.1%) of the participants had never given live birth before the index pregnancy, while 21.5% had given live birth 5 or more times. Nearly one in four women (22.5%) had never attended ANC, while slightly more than one-third (34.4%) of women had attended four or more ANC visits. One in every six women (17.0%) reported IPV during pregnancy for the index pregnancy. Almost half of the participants (47.7%) reported at least one complication experienced during pregnancy. Approximately half of the women (45.3%) gave birth at home for the index pregnancy.

**Table 1.** Characteristics of postpartum women in Ethiopia 2019–2020 (weighted *N* = 2558).

Variables	Categories	Frequency	Percentage
Region	Tigray	176	6.8
	Afar	51	2.0
	Amhara	527	20.4
	Oromia	1120	43.3
	SNNP	581	22.5
	Addis Ababa	103	4.0
Age	<20	272	10.5
	20–24	620	24.0
	25–29	782	30.2
	30–34	467	18.1
	≥35	417	16.1
Residency	Rural	1977	77.3
	Urban	581	22.7
Marital status	Married	2499	97.7
	Others	59	2.3
Educational status	Never educated	1051	41.1
	Primary school	1021	39.9
	Secondary school	300	11.7
	College and above	186	7.3
Wealth status	Poorest	512	20.0
	Poorer	510	19.9
	Middle	511	20.0
	Richer	512	20.0
	Richest	513	20.1
Pregnancy intention	Intended	1633	63.8
	Unintended	925	36.2
Preconception care	Yes	360	14.1
	No	2196	85.9
At least one ANC	Yes	1979	77.3
	No	579	22.7
Adequate ANC (4+)	Yes	879	34.4
	No	1679	65.6
Number of times giving live births	0 (Nulliparous)	461	18.0
	1 (Primiparous)	565	22.1
	2–4 (Multiparous)	982	38.4
	≥5 (Grand multiparous)	548	21.5
Multivitamin-containing iron intake	Yes	1671	65.3
	No	887	34.7
IPV during pregnancy (*n* = 2499)	Yes	426	17.0
	No	2073	83.0
MWH use	Yes	379	14.6
	No	2179	85.4
Place of childbirth	Home	1163	45.5
	Health facility	1395	54.5
Mode of childbirth	Vaginal	2418	94.6
	Caesarean section	140	5.4
Complications during pregnancy	Yes	1220	47.7
	No	1311	51.3
	Don’t know	27	1.0

Abbreviations: ANC-Antenatal Care, MWH-Maternity Waiting Home, IPV-Intimate Partner Violence, Others-Single/divorced/widowed.

### Intrapartum obstetric complications

For analysis of intrapartum obstetric complications, 2545 weighted women were included. The prevalence of intrapartum obstetric complications was 37.6% (95% CI: 35.7–39.5). Figure 2(A) presents the prevalence of intrapartum and specific obstetric complications.

**Figure 2.**
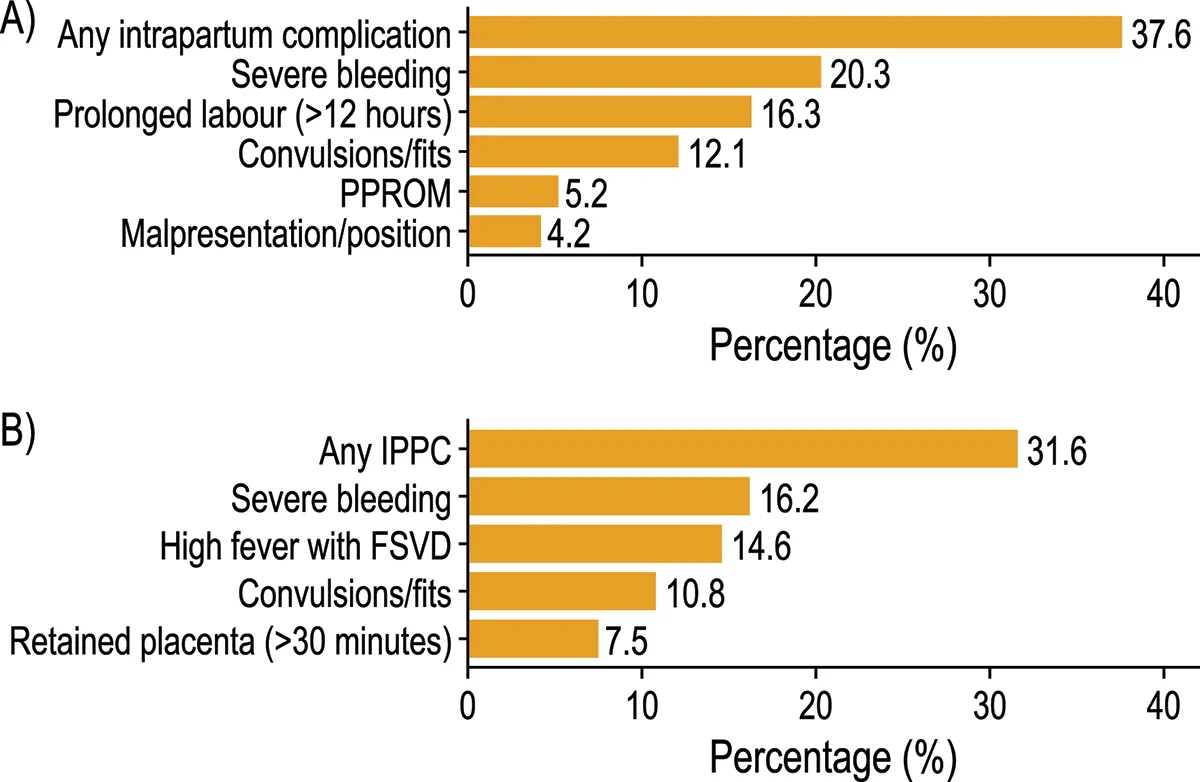
Prevalence of self-reported obstetric complications among women in the PMA Ethiopia longitudinal survey 2019–2020. Panel (A) shows intrapartum obstetric complications, and panel (B) shows immediate postpartum obstetric complications.

### Immediate postpartum obstetric complications

A total of 2,531 women were included for analysis of immediate postpartum obstetric complications. The overall immediate postpartum complication prevalence was 31.6% (95% CI: 29.7–33.4). Figure 2(B) presents the prevalence of immediate postpartum complications and specific complications.

### Factors associated with intrapartum obstetric complications

As presented in Table 2, variables included in the final model, five independent variables, such as residency, parity, IPV during pregnancy, complications during pregnancy, and place of childbirth, remained associated with intrapartum obstetric complications. Women residing rural in areas had a 72% higher prevalence of intrapartum complications (aPR = 1.72,95% CI: 1.34–2.20, *p* < 0.001) compared to their urban counterparts. Nulliparous (no prior live births) women had a 30% higher prevalence of intrapartum obstetric complications (aPR = 1.30,95% CI: 1.07–1.58, *p* = 0.006), and primiparous women had a 20% higher prevalence of intrapartum complications (aPR = 1.20,95% CI: 1.06–1.36, *p* = 0.003) compared with multiparous (who gave live births 2–4 times) women. Women who experienced IPV during index pregnancy had a 31% higher prevalence of intrapartum obstetric complications (aPR = 1.31,95% CI: 1.14–1.50, *p* < 0.001) compared with those who did not experience IPV. Women who experienced complications during pregnancy had a 78% higher prevalence of intrapartum obstetric complication (aPR = 1.78,95% CI: 1.55–2.06, *p* < 0.001) compared with those who did not experience complications during pregnancy.

**Table 2.** Factors associated with intrapartum obstetric complications among postpartum women in Ethiopia (*N* = 2,545).

Variables	Null model	Model 2	Model 3	Model 4	
		aPR (95%CI)	aPR (95%CI)	aPR (95% CI) P- value	
Age					
<20		0.84 (0.66,1.07)		0.87 (0.68,1.09)	0.245
20–24		1.06 (0.90,1.24)		1.08 (0.92,1.27)	0.308
25–29		1		1	
30–34		1.17 (0.98,1.40)		1.15 (0.96,1.38)	0.103
>35		1.09 (0.90,1.32)		1.04 (0.86,1.27)	0.648
Education					
Never		1.03 (0.81,1.29)		1.02 (0.81,1.28)	0.840
Elementary		1.23 (1.00,1.50)		1.21 (0.99,1.49)	0.057
Secondary		1.07 (0.86,1.33)		1.04 (0.84,1.28)	0.699
College & above		1		1	
Wealth status					
Poorest		1		1	
Poorer		0.91 (0.75,1.11)		0.93 (0.77,1.16)	0.471
Middle		0.85 (0.69,1.05)		0.87 (0.71,1.08)	0.209
Richer		0.79 (0.63,0.98)		0.88 (0.71,1.09)	0.262
Richest		0.76 (0.60,0.97)		1.18 (0.88,1.59)	0.263
Number of times give live births					
0 (Nulliparity)		1.32 (1.09,1.60)		1.30 (1.07,1.58)	0.006
1 (Primiparity)		1.19 (1.05,1.34)		1.20 (1.06,1.36)	0.003
2–4 (Multiparity)		1		1	
>5 (Grand multiparity)		1.04 (0.84,1.28)		1.06 (0.86,1.30)	0.578
Preconception care					
no		1		1	
Yes		0.98 (0.83,1.14)		0.97 (0.83,1.14)	0.784
Pregnancy intention					
Unintended		1.08 (0.95,1.24)		1.11 (0.97,1.24)	0.111
Intended		1		1	
Adequate ANC					
no		1			
Yes		1.02 (0.90,1.17)		1.03 (0.90,1.17)	0.630
Multivitamin intake					
no		1		1	
Yes		0.90 (0.78,1.03)		0.89 (0.77,1.02)	0.102
IPV during pregnancy					
no		1		1	
Yes		1.33 (1.17,1.52)		1.31 (1.14,1.50)	<0.001
MWH use					
No		1		1	
Yes		1.19 (1.03,1.36)		1.14 (0.97,1.34)	0.092
Pregnancy complication					
No		1		1	
Yes		1.78 (1.54,2.06)		1.78 (1.55,2.05)	<0.001
Place of childbirth					
Home		1		1	
Health facility		1.58 (1.35,1.85)		1.60 (1.38,1.87)	<0.001
Region					
Tigray			0.94 (0.68,1.30)	0.89 (0.66,1.19)	0.446
Afar			0.45 (0.25,0.78)	0.60 (0.35,1.03)	0.069
Amhara			1.05 (0.78,1.41)	1.01 (0.77,1.34)	0.891
Oromia			0.80 (0.60,1.07)	0.76 (0.58,1.00)	0.055
SNNP			0.91 (0.66,1.27)	0.92 (0.67,1.26)	0.626
Addis Ababa			1	1	
Residency					
Urban			1	1	
Rural			1.22 (1.00,1.50)	1.72 (1,34,2.20)	<0.001
Community MWH use					
low			1	1	
High			1.22 (1.02,1.47)	1.01 (0.85,1.21)	0.822
Random effect					
Variance (SE)	0.15 (0.03	0.09 (0.03)	0.12 (0.03))	0.07 (0.02)	
ICC percentage	13.0	8.3	11.0	6.5	
MPR	1.15	1.07	1.12	1.06	
Model fitness					
Log-likelihood ratio	−1857	−1785	−1847	−1774	
AIC	3719	3620	3713	3613	

Abbreviations: ANC- Antenatal Care, IPV-Intimate Partner Violence, MWH-Maternity Waiting Home.

### Factors associated with immediate postpartum obstetric complications

In Table 3, the final model shows that six independent variables remain associated with the immediate postpartum obstetric complications. These include maternal age, complications during pregnancy, prolonged labour, severe intrapartum bleeding, intrapartum convulsions, and place of childbirth. Women aged 35 years and older had an 18% higher prevalence of immediate postpartum complications compared to women aged 25–29 years (aPR = 1.18, 95% CI: 0.97–1.44, *p* = 0.084), while women aged < 20 years had a 22% lower prevalence of immediate postpartum complications (aPR = 0.78, 95% CI: 0.60–1.01, *p* = 0.069). There was borderline evidence of association, suggesting these findings may require further investigation. Women residing in rural areas had a 32% higher prevalence of immediate postpartum complications (aPR = 1.32,95% CI: 0.99–1.74, *p* = 0.053) compared to their urban counterparts. Women who had given five or more live births (grand multiparity) had an 18% lower prevalence of immediate obstetric postpartum complications than those women who had 2–4 live births (aPR = 0.82, 95% CI: 0.67–0.99, *p* = 0.043), indicating the protective role of high parity. This finding may need further research. Women who experienced complications during pregnancy had a 65% higher prevalence of immediate postpartum obstetric complications compared with women who did not experience pregnancy complications (aPR = 1.65, 95% CI: 1.40–1.93, *p* < 0.001). Women who gave birth in a health facility had a 17% lower prevalence of immediate postpartum obstetric complications (aPR = 0.83, 95% CI: 0.70–0.98, *p* = 0.034). Women who experienced prolonged labour had a 34% higher prevalence of immediate postpartum obstetric complications (aPR = 1.34, 95% CI: 1.14–1.57, *p* < 0.001). Women who had severe vaginal bleeding during the intrapartum period had 2.62 times prevalence of immediate postpartum obstetric complications (aPR = 2.62, 95% CI: 2.20–3.11, *p* < 0.001) compared with women who did not experience severe bleeding during intrapartum. Women who experienced intrapartum convulsions/fits had a 70% higher prevalence of immediate postpartum obstetric complications (aPR = 1.70, 95 % CI: 1.47–1.96, <0.001) compared with those who did not experience intrapartum convulsions.

**Table 3.** Factors associated with immediate postpartum complications among postpartum women in Ethiopia.

	Null model	Model 2	Model 3	Model 4	
Variables		aPR (95%CI)	aPR (95%CI)	aPR (95%CI)	*p*-value
Age					
<20		0.77 (0.59,1.01)		0.78 (0.60,1.01)	0.069
20–24		0.96 (0.81,1.15)		0.97 (0.82,1.16)	0.804
25–29		1		1	
30–34		1.16 (0.97,1.38)		1.15 (0.96,1.37)	0.120
≥35		1.21 (0.99,1.47)		1.18 (0.97,1.44)	0.084
Education status					
Never		1.02 (0.79,1.33)		1.02 (0.78,1.32)	0.879
Elementary		1.07 (0.84,1.37)		1.06 (0.83,1.36)	0.607
Secondary		1.00 (0.76,1.32)		0.98 (0.74,1.28)	0.887
College and above		1		1	
Wealth status					
Poorest		1		1	
Poorer		0.87 (0.71,1.06)		0.87 (0.72,1.07)	0.204
Middle		0.95 (0.80,1.13)		0.95 (0.80,1.13)	0.607
Richer		1.01 (0.82,1.23)		1.06 (0.86,1.30)	0.532
Richest		1.02 (0.82,1.27)		1.29 (0.97,1.72)	0.076
Number of times give live births					
0 (Nulliparity)		0.85 (0.69,1.04)		0.84 (0.69,1.03)	0.111
1 (Primiparity)		0.97 (0.82,1.16)		0.99 (0.83.1.18)	0.925
2–4 (Multiparity)		1		1	
≥5 (Grand multiparity)		0.81 (0.67,0.97)		0.82 (0.67,0.99)	0.043
Adequate ANC					
no		1		1	
Yes		1.03 (0.89.1.19)		1.02 (0.89,1.18)	0.687
Multivitamin intake					
no		1		1	
Yes		0.90 (0.79,1.03)		0.89 (0.78,1.03)	0.134
IPV during pregnancy					
No		1		1	
Yes		1.03 (0.90,1.17)		1.01 (0.88,1.46)	0.810
Complications during pregnancy					
No		1		1	
Yes		1.64 (1.40,1.96)		1.65 (1.40,1.93)	<0.001
MWH					
No		1		1	
Yes		0.99 (0.83,1.17)		0.93 (0.76,1.14)	0.929
Severe bleeding during birth					
No		1		1	
Yes		2.66 (2.24,3.15)		2.62 (2.20,3.11)	<0.001
PPROM					
No		1		1	
Yes		1.08 (0.85,1.37)		1.09 (0.86,1.38)	0.510
Malposition					
No		1		1	
Yes		0.97 (0.80,1.18)		0.97 (0.80,1.18)	0.830
Prolong labour					
no		1		1	
Yes		1.35 (1.15, 1.59)		1.34 (1.14,1.57)	<0.001
Convulsions/fits					
No		1		1	
Yes		1.70 (1.47,1.96)		1.67 (1.44,1.93)	<0.001
Place of childbirth					
Home		1		1	
Health facility		0.81 (0.68,0.96)		0.83 (0.70,0.98)	0.034
Region					
Tigray			1.11 (0.71,1.71)	1.65 (0.81,1.66)	0.396
Afar			0.56 (0.33,0.96)	0.73 (0.46,1.17)	0.196
Amhara			1.28 (0.87,1.89)	1.12 (0.82,1.54)	0.460
Oromia			0.97 (0.65,1.46)	0.96 (0.70,1.31)	0.847
SNNP			1.06 (0.70,1.61)	1.06 (0.74,1.50)	0.740
Addis Ababa			1	1	
Residency					
Urban			1	1	
Rural			1.45 (1.15,1.83)	1.32 (0.99,1.74)	0.053
Community MWH use					
low			1	1	
High			1.10 (0.86,1.40)	1.01 (0.83,1.22)	0.896
Random effect					
Variance of intercept	0.23 (0.05)	0.06 (0.03)	0.2 (0.04)	0.05 (0.02)	
ICC percentage	19	5.7	17	4.8	
MPR	1.24	1.05	1.20	1.04	
Model comparison					
Log-likelihood ratio	−1678	−1496	−1668	−1492	
AIC	3360	3052	3355	3059	

Note: Reported *p* value is from Model 4.

Abbreviation: PPROM – Premature and Prolonged Rupture of Membranes.

### Random effects and model comparison

In Tables 2 and 3, we present the random effects and model comparison parameters. In the null model of intrapartum complications, ICC was 0.130, indicating that 13.0% of intrapartum complications were attributed to cluster membership; the remaining 87.0% was attributed to individual differences among participants.

The prevalence of intrapartum obstetric complications, the empty model fit (AIC = 3719), the model fit improvement in subsequent models, and Model 4 (AIC = 3613) were the best fit. For immediate postpartum obstetric complications, Model 4 (AIC = 3059) is close to Model 2 (AIC = 3052) but has a slightly higher AIC, regardless of the small difference, Model 4 was chosen to maintain the purpose of multilevel modelling. Thus, the interpretation of factors associated with immediate postpartum obstetric complications was based on Model 4.

## Discussion

This study aimed to assess the prevalence and factors associated with intrapartum and immediate postpartum obstetric complications using population-level data in Ethiopia. This evidence on obstetric complications, categorised by timing (intrapartum versus immediate obstetric postpartum) and corresponding associated factors, provides actionable insight into targeted interventions.

### Key findings

The present study found that the self-reported prevalence of intrapartum complications among Ethiopian women was 37.6%, while the prevalence of immediate postpartum obstetric complications (within 24 hours after childbirth) was 31.6%. In this study, several factors were associated with intrapartum and immediate postpartum obstetric complications. Notably, some factors varied by the timing of obstetric complications (intrapartum vs immediate postpartum). Low parity was associated with a higher prevalence of intrapartum obstetric complications but not immediate postpartum obstetric complications. Women residing in rural areas had a higher prevalence of both intrapartum and immediate postpartum complications. Our findings also indicated that IPV during pregnancy was positively associated with the prevalence of intrapartum complications; however, its significant association did not extend into the immediate postpartum period. These findings provide insight into how measuring all obstetric complications during pregnancy, childbirth, and the postpartum period as a single outcome may obscure estimates of associated factors. Women who experienced complications during pregnancy had a higher prevalence of immediate postpartum obstetric complications compared with women who did not experience pregnancy complications. Women who experienced complications during pregnancy had a higher prevalence of both intrapartum and immediate postpartum obstetric complications. In this study, women who gave birth in a health facility had a lower prevalence of immediate postpartum obstetric complications. Women who experienced intrapartum complications (severe vaginal bleeding, prolonged labour, convulsions) were independently positively associated with the prevalence of immediate postpartum complications.

### Comparison with previous evidence

Consistent with our findings, a study in southern Ethiopia reported that 38.8% of women experienced at least one childbirth obstetric complication [27]. The finding is also consistent with a previous study in Kenya, where 35.3% of ANC users experienced labour complications [43]. Similar results were also reported from a population-based cluster randomised trial and a cross-sectional study in Bangladesh [22,28]. Similarly, a population-based prospective study across eight countries in South Asia and SSA found that 33% of women experienced at least one obstetric complication [44]. However, this finding is lower compared with a study in Kenya (42.5%), where experienced labour complications among women classified as high risk at first ANC [43]. The possible reasons for the discrepancy with the study in Kenya were that the study populations were high-risk women, which, by definition, increases the risk of childbirth complications.

These findings underscore the burden of obstetric complications in low-resource settings and highlight the need for targeted interventions to improve both intrapartum and immediate postpartum care. Failure to intervene early and appropriately in obstetric complications may contribute to a high potential of life-threatening complications and maternal deaths [45].

The high prevalence of immediate postpartum complications highlights the need for providers to adhere to the World Health Organisation-recommended immediate postpartum care monitoring and for women giving birth at home to have a postpartum check-up within the first 24 hours after childbirth [46,47]. Women who missed the opportunity to receive a postpartum check-up within 3 hours after giving birth are more likely to develop immediate postpartum complications [48]. Women should be monitored four times during the first hour after childbirth, then hourly and every 4 hours until 24 hours after childbirth [49]. However, in LMICs, among women who give birth in health facilities, only 66% reported that they had been monitored at least once during the 24 hours after birth, and providers do not follow the postpartum guidelines [50,51]. In LMICs, once women give birth, postpartum monitoring is the most neglected aspect of maternity care [50]. Healthcare providers in referral hospitals often fail to follow postpartum guidelines in risk assessment and preventive care for postpartum haemorrhage [51]. All of these may contribute to high immediate postpartum complications. Preventive interventions, such as the provision of uterotonic drugs and active third-stage management, are proven interventions for the prevention of postpartum haemorrhage, but there is an implementation gap in Ethiopia [52].

The current findings are in line with previous reports findings that 30% of women experienced at least one immediate postpartum complication in southern Ethiopia [27]. In a facility-based study, 30.4% experienced complications after caesarean delivery [53]. Similarly, in South Asia and SSA, 32.7% of women experienced at least one direct maternal morbidity [44]. However, the prevalence in Ethiopia is higher than that in Uganda (14%), among women who gave birth in health facilities [48]. The prevalence of immediate postpartum complications is lower than the findings reports from Pakistan, where 36.3% experienced postpartum complications [54]. The difference is expected because the Pakistan study measured postpartum complications over a 6‑week period, whereas our study captured only immediate postpartum period. When complications are measured across long timeframe, more women have opportunity to experience postpartum complications, resulting in a higher reported prevalence.

### Interpretation of associated factors

Our data indicate that rural residence was positively associated with the risk of intrapartum and immediate postpartum complications. This is consistent with findings in India, where the odds of childbirth complications increased among rural women [55]. Contrary to our findings for Northwest Ethiopia, urban women are at a higher risk of pregnancy-related complications [21]. Rural women often face delays in reaching health facilities and limited emergency obstetric care services. Additionally, rural women were less likely to recognise danger signs early and seek appropriate healthcare.

This study found that IPV during pregnancy was significantly associated with an increased likelihood of intrapartum complications, with women who experienced IPV being 31% more likely to encounter complications during childbirth. This aligned with a previous study, which indicates IPV during pregnancy increases the risk of childbirth complications such as intrapartum bleeding, leakage of membranes, and antepartum bleeding [56]. This association is biologically and socially plausible, as IPV increases maternal stress, reduces women’s autonomy, and their ability to seek care during illness [57,58]. Stress and physical trauma related to IPV during pregnancy may also contribute to physiological dysregulation, which can increase vulnerability to obstetric complications. Despite the negative health consequences of IPV, most cases of IPV in Ethiopia are not disclosed, leaving women unable to access tailored care, and counselling services nor police protection [59]. These findings underscore the importance of integrating IPV screening and support within ANC to mitigate preventable complications during childbirth [46].

The findings showed that complications during pregnancy were positively associated with a higher prevalence of both intrapartum and immediate obstetric complications. This is consistent with findings reported from several studies that indicate that pregnancy complications increase the risk of childbirth complications [22,55]. This pregnancy complication association with both intrapartum and immediate postpartum complications is consistent with the continuum of maternal morbidity; poorly managed antenatal complications, such as hypertensive disorders of pregnancy, infections, or antepartum haemorrhage, can enhance vulnerability during the postpartum period. The presence of antepartum haemorrhage increases the risk of primary postpartum haemorrhage [19,60]. This is because causes of antepartum bleeding, such as placental abruption and placenta previa, increase the risk of PPH [18]. These provide insight into the need for early identification and prevention of complications during pregnancy to reduce intrapartum complications. However, evidence from four countries (Ethiopia, South Africa, Kenya, and India) indicates gap in pregnancy risk identification during ANC and in providing further management [61]. The same study indicates that women with complaints of obstetric danger signs did not get further care during pregnancy. Only 6% of women with complaints of obstetric complications or high risk were referred to a hospital or specialised care [61], a gap like this may contribute to high intrapartum complications. Evidence from low-income countries shows that women who reported pregnancy danger signs received the same care as women without complication danger signs [62]. Poorly managed antenatal complications can heighten physiological vulnerability during labour and delivery. Additionally, women with complications during pregnancy may require more frequent monitoring and timely intervention, which can alleviate intrapartum complications. These imply that efforts to identify pregnancy complications and provide appropriate interventions for those experiencing complications during pregnancy are required; likewise, this may help to reduce complications during childbirth. Thus, multimodal screening and management for pregnancy complications are required to prevent, identify, and treat complications during pregnancy, which may prevent further complications at childbirth and postpartum [63].

The results show that nulliparity and primiparity were associated with a higher prevalence of intrapartum complications compared to multiparity (parity 2–4). This aligned with the study in Bangladesh, where first-time mothers have high odds of childbirth complications [22]. Evidence shows notable differences in obstetric complications between nulliparous and multiparous women. Women without prior birth have increased odds of prolonged labour, preeclampsia and premature rupture of membranes [64]. When the uterus contracts for the first time during labour, this often results in a longer latent and active phase, a longer second stage, and a more prolonged labour. Additionally, first-time women face more cervical resistance and higher prevalence of prolonged labour. Nulliparity with advanced maternal age increases the risk of gestational hypertension and premature rupture of membranes, while grand multiparity increases the risk of gestational diabetes and preterm birth [64]. However, a study in Ethiopia reported no difference in obstetric outcomes by parity [17], suggesting that the association between parity and combined obstetric outcomes may be confounded by other unknown factors.

Prolonged labour was positively associated with a higher prevalence of immediate postpartum obstetric complications. This is consistent with previous studies, which reported that prolonged labour increases the risk of postpartum haemorrhage [18–20]. This could be prolonged labour, increasing the risk of induction of labour and instrumental delivery, which imposes a high risk for postpartum complications. Additionally, prolonged labour causes perineal tears and uterine atony, which contribute to immediate postpartum complications. These imply that women with suspected slow progress of labour need to be evaluated early, and appropriate intervention should be initiated based on the underlying cause to prevent prolonged labour. Furthermore, the findings suggested that women with prolonged labour require closer monitoring in the immediate postpartum period. These all help prevent complications during childbirth and the postpartum period.

This study showed that severe vaginal bleeding during intrapartum was strongly associated with higher prevalence of immediate postpartum obstetric complications. This finding is clinically plausible, as heavy intrapartum bleeding can lead to haemodynamic instability, reduced uterine tone, and depletion of maternal reserves, all of which increase vulnerability to postpartum complications. Additionally, this may be explained by causes of intrapartum bleeding, such as placental abruption, placenta previa, and placenta accreta spectrum, which also cause postpartum bleeding [18]. These findings highlight the critical need for timely recognition and management of obstetric emergencies during labour to prevent complications after childbirth. Also, women with severe intrapartum bleeding need more attention during the postpartum period.

In this study, we found that facility childbirth is positively associated with the prevalence of intrapartum complications. Consistent findings reported in studies in LMICs that maternal morbidity is high when women give birth in a hospital compared to giving birth in a non-hospital setting [65]. Additionally, a study in India based on the National Family Health Survey found that the odds of complications are higher among women who give birth at a health facility than at home [55]. This may be explained by 1) Women with high-risk pregnancies are more likely to be referred to health facilities, increasing the odds of childbirth complications in these facilities [43]; and 2) Self-reported maternal complications increase with improving maternal health services through enhancing reporting behaviour. Findings in India reported that facility-based childbirth contributed to 13% of complications, due to improved reporting [66], which means facility childbirth increases women’s ability to report obstetric complications [66]. Women giving birth at home might not recognise complications during labour and childbirth. These findings should be interpretation requires caution. Giving birth in a health facility does not increase the risk of childbirth complications. Instead, low-risk women tend to give birth at home, while high-risk women give birth at health facilities. Also, reporting of complications improves with childbirth in health facilities because healthcare providers may identify some complications and inform women, and women could be aware of and report them. These include malposition and presentation.

This study found that births at health facilities were associated with a 17% lower prevalence of immediate postpartum obstetric complications. This protective role is consistent with skilled attendance at birth, timely recognition of danger signs, and access to emergency obstetric care, which reduce postpartum complications. Enhancing health facility childbirth coverage may significantly reduce immediate postpartum complications. These all imply the need to strengthen monitoring and the quality of intrapartum and immediate postpartum care, thereby reducing the burden of immediate postpartum obstetric complications. A comprehensive assessment of all labouring women by skilled birth attendants at health facilities enables early identification of women at risk of intrapartum and postpartum complications, accompanied by timely and appropriate intervention, which can reduce intrapartum and postpartum obstetric complications [46]. A pre-post implementation study in Ethiopia shows that the WHO safe childbirth checklist for intrapartum care enhances health care provider adherence to essential birth care [67]. Likewise, the WHO safe childbirth checklist reduces both maternal morbidity and perinatal deaths [68,69]. Furthermore, implementing the WHO safe childbirth checklist improves the quality of preeclampsia and infection management, as well as postpartum counselling and evidence-based practice [68,69]. However, evidence shows implementation of the WHO safe childbirth checklist for intrapartum care in Ethiopia remains inadequate [70]. Additionally, evidence indicates that individualised care for high-risk women improves maternal and foetal outcomes [63]. In settings where homebirth is common, emphasis on the need for self-monitoring strategies in the immediate postnatal period, including recognition of symptoms like seizures, heavy bleeding and changes in consciousness, and promoting early postnatal check-ups may help prevent further postpartum complications. Strengthening maternity home use for high-risk pregnant women could reduce obstetric complications during labour and the immediate postpartum period [71]. However, in the present study, use of maternity homes was not significantly associated with either intrapartum or immediate postpartum obstetric complications.

### Implications of the findings

The findings highlight the need for tailored interventions to address specific associated factors for intrapartum and immediate postpartum complications and ultimately to improve maternal outcomes. The consistently high prevalence of both intrapartum and immediate postpartum complications among rural women highlights the need to improve access to skilled birth attendance, emergency obstetric care, and timely referral systems in rural settings. Findings suggest strengthening early detection and recognition of complications during pregnancy, followed by further obstetric/medical management to reduce complications during the intrapartum and immediate postpartum period. The findings suggest that women who experience pregnancy complications during the index pregnancy need close follow-up during childbirth and the postpartum period. Integrating IPV during pregnancy and support into ANC, followed by mitigation strategies and more attention during intrapartum care for those who experienced IPV during their pregnancy, may reduce the burden of obstetric complications. Reinforcing the importance of facility childbirth attendance in preventing postpartum complications through community mobilisation may reduce immediate postpartum complications. Strengthening labour monitoring through the implementation of the WHO safe childbirth checklist may reduce intrapartum and immediate postpartum obstetric complications. For nulliparous and primiparous women, tailored counselling and risk communication should be integrated into ANC and birth preparedness and complication readiness interventions. The findings highlight differences in some factors associated with intrapartum and immediate postpartum complications. Future research should focus on factors associated specific to pregnancy complications, labour complications, and postpartum complications instead of factors associated with overall obstetric complications. Future longitudinal studies should include objective assessment of intrapartum and immediate postpartum complications by trained healthcare professionals to improve the accuracy and reliability of complication measurement at population level.

### Strengths and limitations

The main strength of the study is the use of nationally representative data from five regions and one city administration, which includes both women who gave birth at home and those who gave birth in health facilities. We used advanced statistical analysis and sample weighting to account for sampling complexity. We also employed a stratified analysis of intrapartum and immediate postpartum obstetric complications, which identifies associated factors specific to each timing of complication (intrapartum and immediate postpartum), providing insight for targeted intervention recommendations.

However, this study has several limitations. First, the use of self-reported obstetric complication symptoms may introduce social desirability bias, recall bias, and an inability to objectively measure complications; measures taken to minimise these issues included asking women multiple tailored questions, and an interview was conducted within a reasonable time following childbirth, 6 weeks after childbirth. Furthermore, the ‘do not know’ response option was considered to avoid forcing women to respond either yes or no. Women self-reporting outcomes may also be liable to misclassification of intrapartum or immediate postpartum complications, a measure taken by asking separate questions for each period of time, both intrapartum and immediate postpartum. Second, a composite outcome measure was employed, which may affect estimates of factors associated with the outcome, as factors associated with different obstetric complications may differ. To address this, we stratified obstetric complications into intrapartum and immediate postpartum to minimise bias in measuring the composite.

## Conclusion

This study found a high proportion of women in Ethiopia experienced intrapartum and immediate postpartum obstetric complications, underscoring the need for strengthening prevention, early detection, and management of obstetric complications. Some factors associated with intrapartum and immediate postpartum complications differ, indicating that these two critical periods are influenced by distinct risk profiles. Nulliparity and primiparity elevate the prevalence of intrapartum complications. Complications during pregnancy were positively associated with the prevalence of both intrapartum and immediate postpartum obstetric complications. IPV during pregnancy is associated with a higher prevalence of intrapartum obstetric complications but not immediate postpartum complications. In this study, most intrapartum complications were positively associated with the prevalence of immediate postpartum complications, while facility childbirth lower prevalence of immediate postpartum complications. Healthcare providers should prioritise enhanced intrapartum monitoring for nulliparous and primiparous women, given their high prevalence of obstetric complications.

## Supplementary Material

STROBE_checklist.docx

Supplementary File.docx

## Data Availability

The data supporting the findings of this study are presented in the main manuscript. In addition, the original data can be obtained from the Performance Monitoring for Action (PMA), which is publicly available from https://www.pmadata.org/data/available-datasets.
