# Supplementary material for: Prevalence and factors associated with intrapartum and immediate postpartum obstetric complications in Ethiopia: a secondary analysis of a national longitudinal survey
Source: Glob Health Action. 2026 Nov 3;19(1):2754625. doi: 10.1080/16549716.2026.2754625 (PMC13761076; doi:10.1080/16549716.2026.2754625)
Supplement: Supplementary file 1 [file ZGHA_A_2754625_SM6980.docx]

**Multilevel modified Poisson regression Stata command syntax**

Model 1: Intercept only model:

meglm outcome_variable [pweight= weighting_variable] || cluster variable:, family(poisson) link(log) vce(robust) eform

Model 2: Individual level variables:

meglm outcome_variable individual_variables [pweight= weighting_variable] || cluster variable:, family(poisson) link(log) vce(robust) eform

Model 3: Community level variables:

meglm outcome_variable community_ variables [pweight= weighting_variable] || cluster variable:, family(poisson) link(log) vce(robust) eform

Model 4: Individual and community level variables:

meglm outcome_variable individual_variables community_ variables [pweight= weighting_variable] || cluster variable:, family(poisson) link(log) vce(robust) eform
